# Supplementary material for: Exploring the Relationship of rs2802292 with Diabetes and NAFLD in a Southern Italian Cohort—Nutrihep Study
Source: Int J Mol Sci. 2024 Sep 1;25(17):9512. doi: 10.3390/ijms25179512 (PMC11394752; doi:10.3390/ijms25179512)
Supplement: Supplementary file 1 [file ijms-25-09512-s001.zip › ijms-3144359-supplementary.pdf]

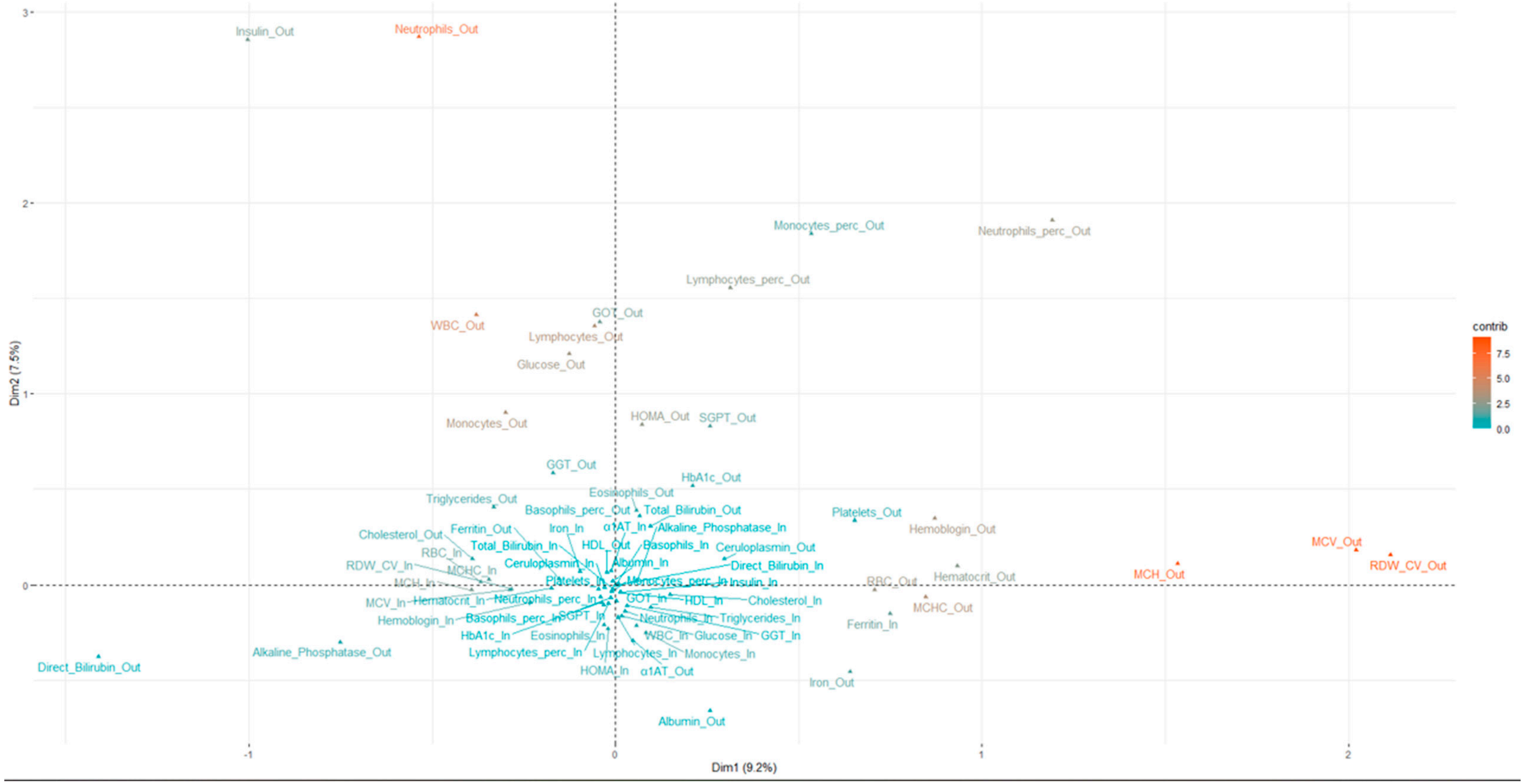

Figure S1. MCA of categorical blood parameters in GG subjects.

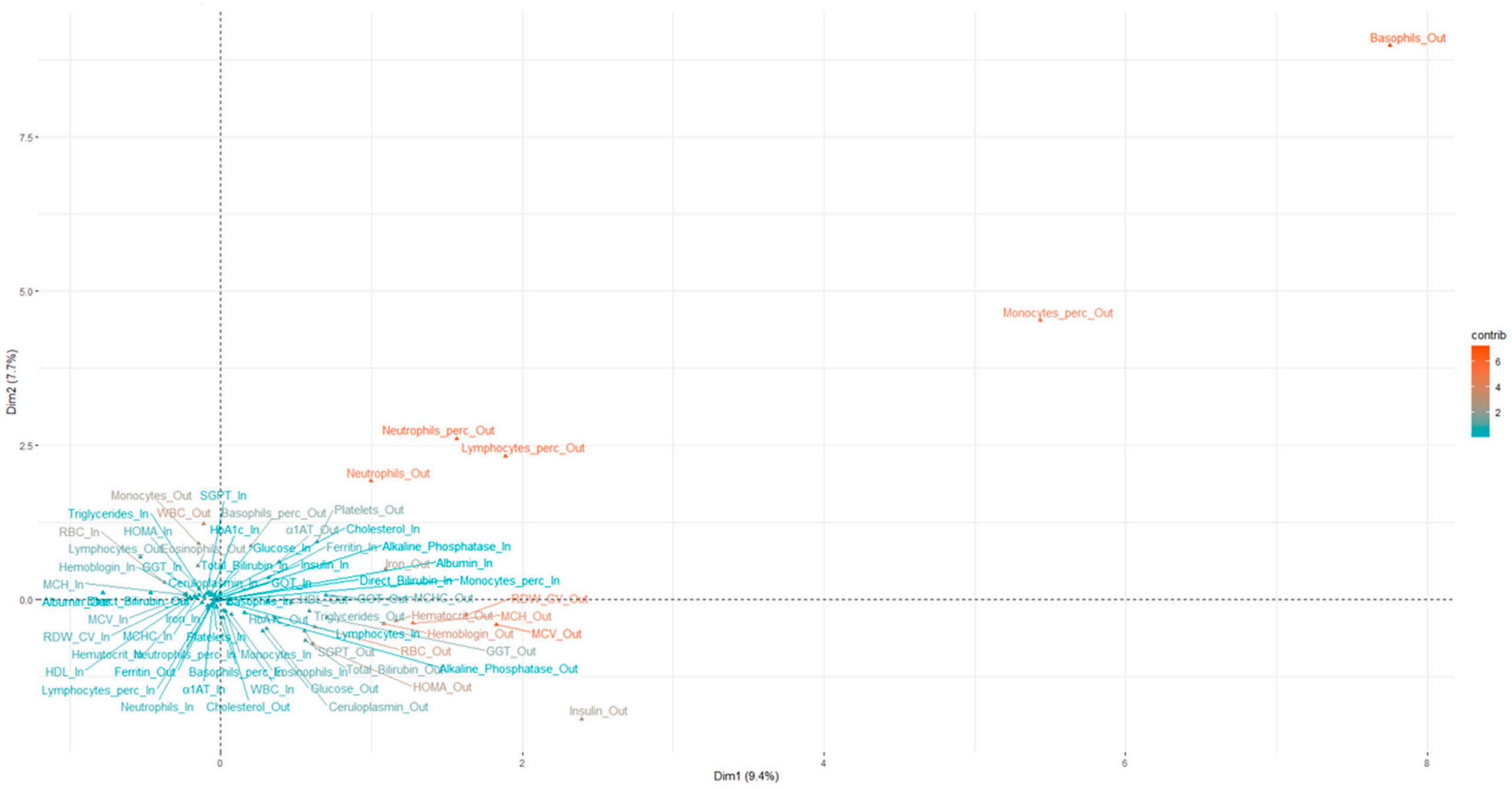

Figure S2. MCA of categorical blood parameters in GT subjects.

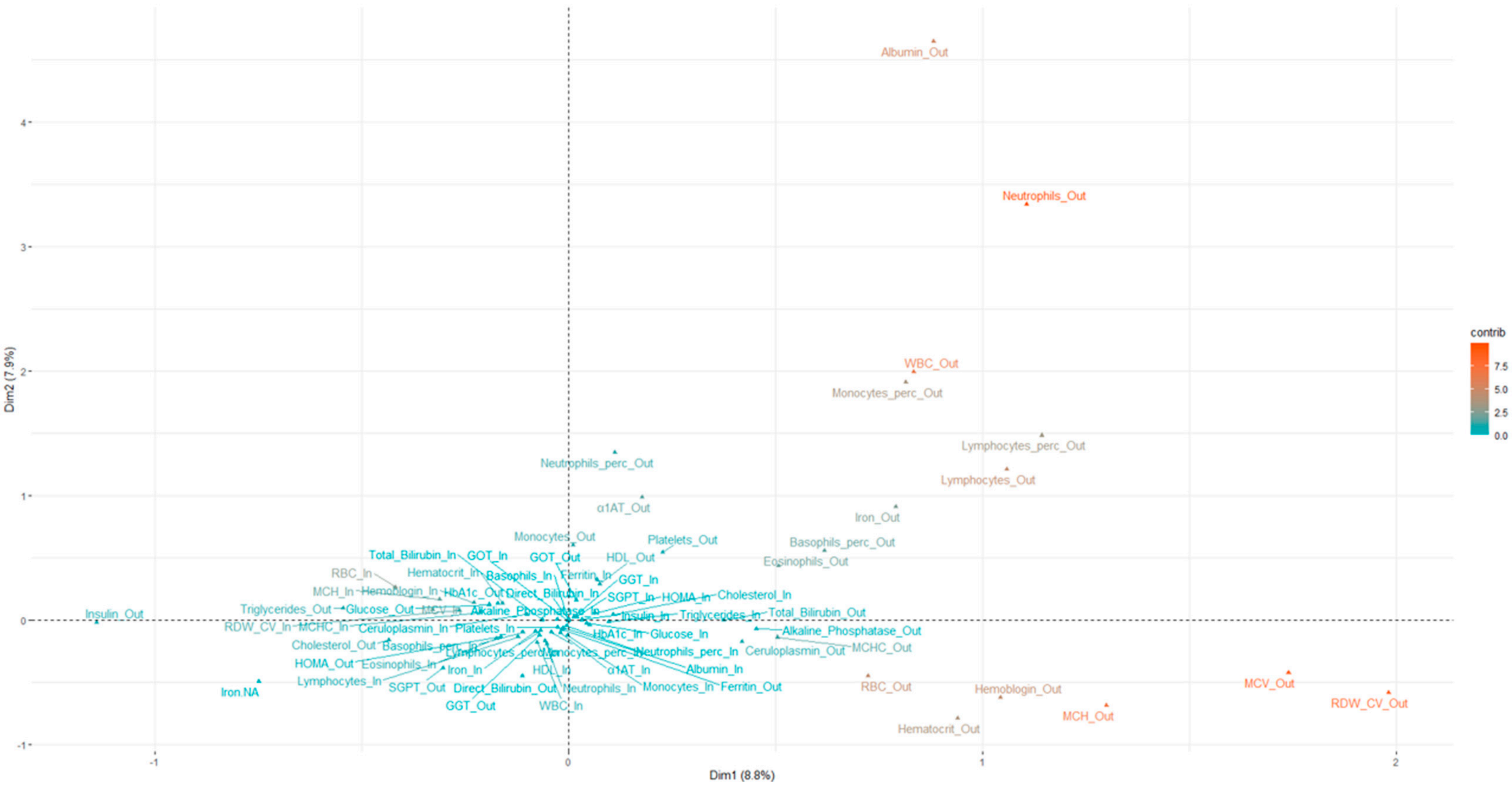

Figure S3. MCA of categorical blood parameters in TT subjects.
